# Performing arts as a non-pharmacological intervention for people with dementia and care-partners: a community case study

**DOI:** 10.3389/fpsyg.2023.1149711

**Published:** 2023-05-09

**Authors:** Laura H. Malinin, Meara Faw, Deana Davalos

**Affiliations:** ^1^Design and Merchandising Department, College of Health and Human Sciences, Colorado State University, Fort Collins, CO, United States; ^2^Communication Studies Department, College of Liberal Arts, Colorado State University, Fort Collins, CO, United States; ^3^Psychology Department, College of Natural Sciences, Colorado State University, Fort Collins, CO, United States

**Keywords:** dementia, psychosocial programs, non-pharmacological interventions, symphony, dance, theater, performing arts, Alzheimer’s disease (AD)

## Abstract

Participation in psychosocial enrichment activities, such as music and arts programming, have shown potential to delay or reduce functional decline - without adverse effects that can be associated with pharmaceuticals. The performing-arts programming described in this community case study was inspired by a community music program called B-Sharp Music Wellness, located in Phoenix, Arizona, which involved small groups of musicians who provided symphony performances for people with dementia. Our community programming sought to engage people with dementia and their informal care partner (typically a spouse) in existing performing-arts programs in their local community, providing social hours and season tickets for either symphony, dance (ballet), or non-musical theater performances. This case study describes the program history and design, including outcomes and lessons learned from the program evaluation of the last full season (2018–19) and partial season (2019–20), when the program was halted due to the COVID-19 pandemic. Program outcomes suggest strategies for, and benefits of, design for performing-arts programs as psychosocial interventions in other communities.

## 1. Introduction

Approximately 55,000,000 people worldwide live with incurable neurodegenerative diseases, including Alzheimer’s and related dementias (ADRD), with roughly 10,000,000 new cases diagnosed per year (World Health Organization, 2022). For some types of dementia (e.g., Alzheimer’s), the disorder may span 15–24 years, including the preclinical, prodromal, and dementia stages (Vermunt et al., 2019). Most of this timespan occurs before institutional care is an option, meaning dementia caregiving can be quite long, with the degree of assistance required increasing over time. Caring for a person with ADRD (PWD) is associated with significant stressors, and growing evidence demonstrates that informal care-partners’ (CPs) needs are often unaddressed by healthcare professionals or PWD interventions (Sörensen and Conwell, 2011; Association, 2019; Lindeza et al., 2020).

For PWDs and CPs, treatment options are limited to sparse choices in non-pharmacological interventions that can require additional coordination, transportation, or fear of stigma for the PWD or CP (Boots et al., 2015). Most pharmacological interventions have major limitations, including poor efficacy, adverse side effects, prohibitive costs, and positive outcomes associated only with the earliest disease stages (Fink et al., 2020; Seibert et al., 2021). Interventions that consider both PWD and CP needs are necessary for optimizing their quality of life (QOL). It is well-established that as CP burdens increase, QOL of both PWD and CP decline (Dauphinot et al., 2015; Olsen et al., 2016). Community-based interventions that include PWD-CP dyads are one option to improve dyadic QOL, minimize stigma, and address specific needs of each person to optimize outcomes. Benefits of these programs are mixed, often a function of the type of activity in which dyads participate. However, a common theme is a sense of reduced CP burden and reduction in agitation and increased engagement in the PWD (Gitlin et al., 2008). Community-based arts programs, specifically, have also been associated with a host of benefits for the PWD, including increased participation, communication, and social cohesion, in addition to improved attention and cognition (Ward et al., 2021).

The B/C-Sharp community-based performing-arts program sought to address many of the current limitations in traditional pharmacological and non-pharmacological interventions. The program intentionally involved CPs, not only to enhance the PWDs’ experiences, but also to enhance CP QOL. Programming took place at a community performing-arts center, capitalizing on a normalized social environment that was dementia-friendly. The program also explored benefits of the dyad-focused intervention across a variety of outcome measures spanning biological, psychological, and cognitive health, providing insights regarding strengths and weaknesses of each outcome measure and enhancing our understanding of how CPs can best be included in future programs. The goal of this manuscript is to outline elements of the B/C-Sharp program as well as detail the evolution of our research approach and lessons learned *via* this multiyear community engaged case study in hopes that others interested in implementing similar programs can learn from our efforts.

## 2. Context

B/C-Sharp began in fall 2015 with a symphony experience. With support from the National Endowment for the Arts, programming expanded in 2018 to include dance and theater as alternative programs and to compare participant perceptions between the three programs. All three programs were offered until March 2020, when B/C-Sharp was halted due to the COVID-19 pandemic. A goal ofB/C-Sharp was to better understand lifestyle or environmental factors benefiting cognitive health and wellbeing in healthy aging and in PWD. This paper focuses on program design, procedures, evaluation methods, and outcomes of the 2018–19 season and partial 2019–20 season. Program evaluation included the “gold standard” measurement (paper and pencil cognitive testing) that is used in traditional pharmacological studies and clinical trials involving older adults to evaluate whether a community-based non-pharmacological intervention could also lead to improved changes in cognition with participants with neurocognitive decline (Buckley et al., 2017). These findings, lessons learned, and best practice recommendations will be addressed.

### 2.1. Setting

B/C-Sharp took place at a performing-arts center in a community of approximately 170,000 individuals (U.S. Census Bureau, 2020). The center featured two performing-arts spaces; five event spaces; and an art gallery. Parking was available on-site and nearby. Symphony and dance performances were held in the Performance Hall (1187-seat proscenium theater), and theater performances took place in the Theater (226-seat proscenium theater). Social hours occurred in one of two ballrooms, which were set up similarly. At the front of the room were a refreshments table, a large table with chairs for socializing, and a small table where participants collected their tickets. Twelve circular tables were spread out in the back half of the room for PWDs’ cognitive assessments and a larger table was off to the side for saliva collection.

### 2.2. Participants

Participants were recruited from senior centers, senior housing communities, churches, local nonprofits, and related organizations, with the goal of recruiting 15 dyads each for the new dance and theater programs.^1^ Recruitment flyers promoted performance dates and descriptions. Inclusion criteria specified pairs of local older adults (age 55+ for PWD) who could attend three or more performances for the season. Dyads included a PWD showing cognitive impairment (per cognitive assessments and/or medical diagnoses) and that person’s CP. Participants attended performances wearing any corrective eyewear and/or hearing devices.

All project elements received institutional review board approval with participants providing written informed consent. A total of 208 unique participants (134 female and 74 males; 98 dyads and 12 healthy controls) enrolled in the program from 2018 to2020, including 34 dyads for dance and 30 dyads for theater. Dyads included 56 spousal/partner, 16 parent/child, 3 friends, 2 professional caregivers, and 1 sibling relationship. Dyad relationships were unknown for participants who began the program prior to 2018. Cognitively healthy controls, added in the 2019–20 season, were not required to participate in dyads. PWD were aged 55–95 years, with 58 having formal ADRD diagnoses. Dates of diagnosis ranged from 1994 to 2018 (*M* = 2013). CPs were 40–91 years old, and healthy controls were 61–81 years old.

### 2.3. Program design and evaluation

**Figure 1:**
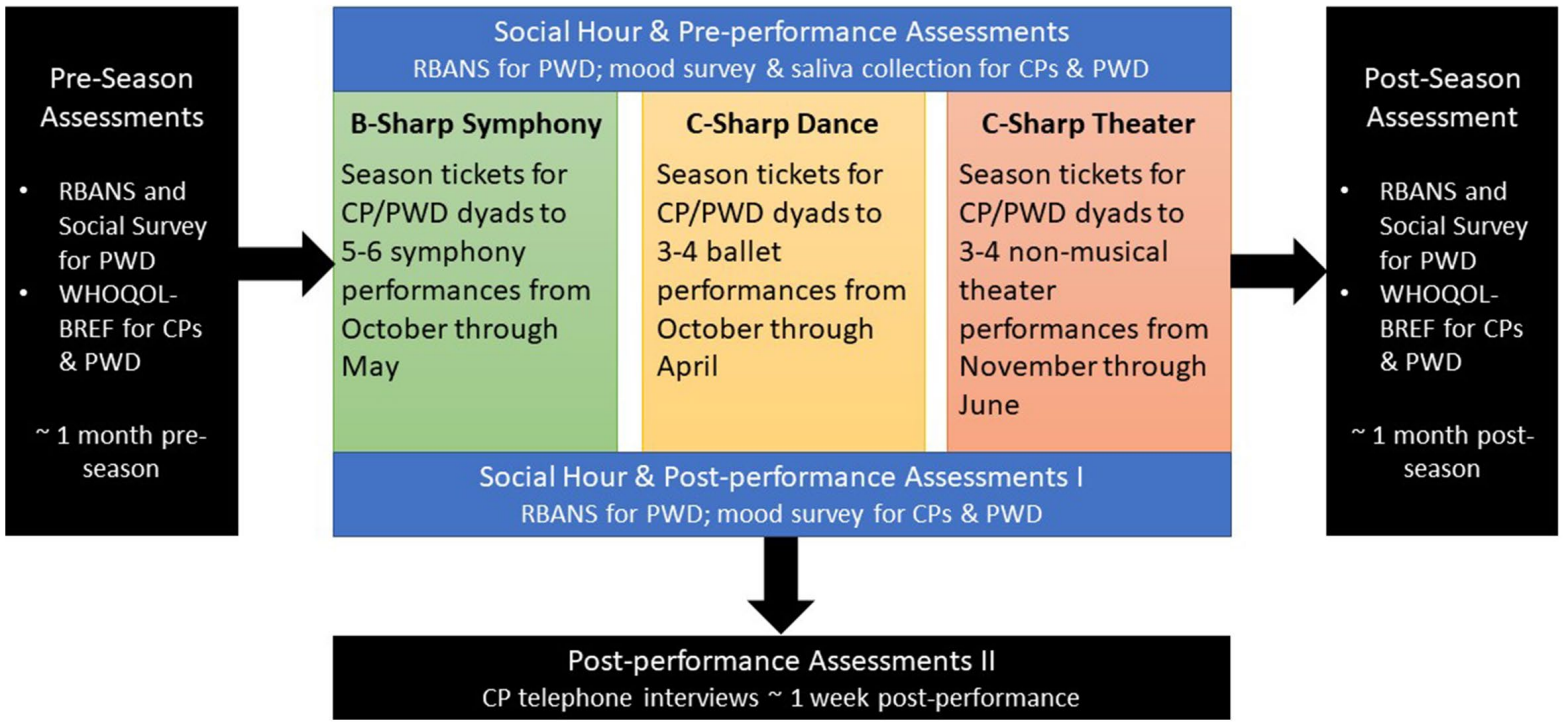
B/C-sharp program design.

Three parallel programs were implemented in fall 2019 (Figure 1), each offering a social hour before and after every performance. Program evaluation involved pre- and post-season assessments, pre- and post-performance assessments, and post-performance CP telephone interviews.

About 1 month prior to each program season, participants attended an intake session. CPs and PWDs (when able) provided information *via* survey about the PWD’s age, gender, educational attainment, career experiences, hobbies/interests, health, cognitive impairment diagnosis, and medications. Dyads also completed a QOL measure developed by the World Health Organization (WHOQOL-BREF). Cognitive performance for PWDs and healthy controls were measured with the Repeatable Battery for the Assessment of Neuropsychological Status (RBANS), a gold standard in the assessment of cognitive decline for its domain specificity, short duration, and high test–retest reliability through the use of parallel testing forms (Randolph et al., 1998). The RBANS allows for assessment of both extended and acute domain changes in cognition. In addition to five RBANS index scores, we calculated 12 standardized subtest scores, allowing for a holistic view of cognitive functioning taken from multiple domains of interest. This assessment was administered by trained undergraduate and graduate students. The full RBANS assessment and the WHOQOL-BREF were completed at intake and again approximately 1 month after the performing-arts season concluded.

Assessments were also conducted during the social hour before and after each performance, with follow-up CP telephone interviews one-week post-performance (Supplementary Appendix A). Pre- and post-performance assessments included RBANS (typically one verbal/auditory RBANS subtest paired with one visuomotor subtest) for PWDs and a mood survey for CPs and PWDs. Saliva was also collected from dyads pre-performance. The collection method (passive drool) was noninvasive, and samples were analyzed for changes in telomere length to assess whether B/C-Sharp engagement resulted in physiological changes indicating enhanced wellbeing. Significant research over the past two decades indicates telomere length is a reliable marker of aging, health, and overall risk of mortality (Cawthon et al., 2003). Additionally, researchers have associated shortening telomere length with risk of developing Alzheimer’s disease (Thomas et al., 2008), increased ADRD progression (Panossian et al., 2003), and susceptibility to chronic health conditions among ADRD CPs (Damjanovic et al., 2007). Recent research suggests the collection and analysis of physiological measures (i.e., salivary cortisol) in arts engagement research is feasible (Bourne et al., 2019), thus we sought to explore the feasibility of collection and analysis of salivary telomere in our study.

Other measures included wearable activity trackers and direct observations. Participants wore Fitbit activity trackers to measure heart rate, activity rate, and sleep patterns before, during, and after attending a performing-arts event. Direct observations were conducted by trained graduate students and recorded in detailed field notes for five events during the 2018–19 season (Supplementary Appendix B). See Table 1 for descriptions of measures, procedures, rationale, key findings, and lessons learned.

**Table 1:** Program evaluation methods, procedures, findings, and lessons learned.

Methods & instruments	Data collection and analysis	Summary of findings	Lessons learned
Repeatable battery for the assessment of neuro-psychological status (RBANS)	PWDs were administered RBANS forms A and B to evaluate cognitive performance pre- and post-season (approximately 10 months apart.) PWDs completed domain-specific subtests pre- and post-performance. For the 2018–2019 season, there was not a significant difference noted between pre-and post-season RBANS scores for the 8 PWD who completed both sessions of testing (*p* < 0.46). For the 2019–2020 season, there was no post-season testing for any participants due to the pandemic	Although not statistically significant, total RBANS scores, on average, improved for PWDs in the symphony program from beginning to end of the season, In terms of acute changes from pre-concert to post-concert, an auditory subtest of the RBANS was typically paired with a visuomotor subtest. Results were not significant, however, general improvement on the auditory subtest was observed. Sample size was too small to analyze dance or theater participants or compare acute RBANS scores between programs	Cognitive paper and pencil tests have been the gold standard for outcome measures for older adults in clinical trials and pharmacological studies. Although a promising method of assessing cognitive impacts, assessments were often frustrating for PWDs and difficult for CPs to see their loved ones struggle. Some participants were motived to participate by the research. The greatest changes associated with program involvement were observed on Total RBANS score pre-season compared to post-season, although less than 10 participants completed all of their pre and post-test assessments necessary for analysis. It may be more useful to stress the importance of completing testing at those two time points rather than at all performances
CP interviews	Phone interviews were conducted with CPs within approximately one week after a performing arts event. Interviews were transcribed by trained graduate students and inductively analyzed by the first author in the NVIVO qualitative analysis software program through a process of open coding, axial coding, and selective coding as well as data visualization. Themes were compared with results from qualitative analysis of interviews from prior years of the symphony program, previously conducted by the second author	CPs felt participation in community performing arts programming was very beneficial for improving mood, memory, social relationships, and overall wellbeing for them and their PWD-partners. Themes were consistent across program types (symphony, dance, or theater)	CP interviews were critical for understanding program benefits and challenges and also for making sense of sometimes confusing data collected through other instruments (e.g., Mood Scale, Activity Trackers)
Mood scale	PWDs and CPs recorded their mood prior and after each event attended. Scales were not statistically analyzed due to small sample size; however they were used in conjunction with interviews to better understand the impact of program participation on mood	The mood scales showed mixed results due to extenuating factors, such as sadness about going home, testing frustration, etc., however they generally improved for both CP and PWD from pre- to post-performance. Analysis of mood scales together with interviews revealed that mood generally improved for dyads days ahead of the performing arts event due to anticipation and stayed elevated several days after the event as participants reminisced	Mood scales may be useful if used to cue interview questions to better understand changes in mood. Mood journals kept by CPs might be helpful to capture mood changes through anticipation (pre-performance) and reminiscence (post-performance)
Observation	Direct observations were conducted by trained graduate students and recorded in detailed field notes during the social hour and performances. Field notes were inductively analyzed by the first author in the NVIVO qualitative analysis software program through a process of open coding, axial coding, and selective coding.	PWDs and CPs were engaged during social hours and performances. Analysis of the physical program setting revealed affordances that interior designs might offer that enhance psycho-social programming and also illustrated challenges that architectural spatial layouts can create for PWCI participation.	Observations for triangulating findings from interviews and mood scale and revealed new insights about person-environment relationships.
Wearable activity tracker	Dyadic pairs wore Fitbit activity trackers to measure heart rate, activity rate, and sleep patterns for 11 consecutive days, beginning five days before attending a performing arts event, the day of the event, and five days after the event. Measures from daily steps were recorded through arm movement, whereas heart rate and sleep were recorded *via* a heart rate monitor on the underside of the tracker. Minutes of sleep per night, average resting heart rate per day, and average steps taken per day were recorded from each participant. Using these data points, we assessed whether these physiological data points changed over the course of symphony attendance.	Results from the pilot indicated some challenges with feasibility. Very few participants (*n* = 2) consistently wore the fitness trackers overnight, making sleep tracking difficult. Four participants wore the trackers consistently before, during, and after the arts event, though no statistically significant differences were observed in daily resting heart rate or average daily steps. This lack of statistically significant findings is likely, in part, due to the small number of participants in the first pilot trial (*n* = 6) as well as the inconsistency in wearing the fitness trackers. That said, results indicate some positive changes (i.e., increased daily step counts the day of the art events) in physiological health associated with attending performances.	CP burden was too high to be useful; participants were frustrated with charging and syncing devices; the database was challenging for the researchers to use.
Telomere length	PWDs and CPs gave salvia samples before each performance attended. The collection methods (i.e., passive drool saliva collection into a plastic tube) are noninvasive and saliva were analyzed to assess changes in telomere length. Samples were assayed by a professional laboratory and then evaluated by a member of the research team.	Results from the 2018–2019 season indicated that telomere lengths did not significantly change for PWDs or CPs in the symphony, dance, or theater groups as evidence *via* paired samples *t* tests (all *p*-values ranged from 0.14 to 0.85). Given that telomeres naturally shorten over time, this stasis could be seen as positive outcome for PWDs and CPs, though additional research would be needed to confirm this conclusion. Interestingly, data did show that telomere lengths significantly differed between PWDs and CPs in the dance (PWD: 1.85; CP: 2.30, *p* < 0.001) and theater (PWD: 1.93; CP: 1.58, *p* < 0.05) groups. There were no statistically significant differences in the symphony groups. Across all programs, the amount of missing data and small participant sample sizes make it difficult to draw any definitive conclusions or to run more complex analyses involving multiple datapoints (i.e., not a pre- and post-test analysis using *t* tests).	Communicating clearly about the purpose of saliva testing was valuable. CPs and PWDs bought into the process. Creating a sense of lightness and humor around the collection also contributed to higher participation rates.
World Health Organizations Quality of Life Instruments (WHOQOL-BREF)	CPs were asked to complete a WHOQOL-BREF for themselves and one on behalf of their PWD-partner pre- and post-season (approximately 10 months apart).	Surveys were not analyzed due to inaccurate data. QOL was assessed through CP interviews.	Although the survey is not long, CPs became confused about whether they were answering for themselves or their loved one, resulting in inaccurate data. Recommend using only one survey and perhaps color-coding surveys to help reduce CP fatigue and confusion.
Social survey	The research team constructed a survey to assess any changes in social engagement and other behaviors related to wellbeing and quality of life. CPs were asked to complete a survey for themselves and one on behalf of their PWD-partner pre- and post-season (approximately 10 months apart)	Surveys were used to collect demographic information only. They were not statistically analyzed due to incomplete and inaccurate data. Some social data was collected through CP interviews	CP burden was too high. Surveys had too many questions and CPs became confused about whether they were answering for themselves or their partners resulting in incomplete and inaccurate data. Survey collection at performing-arts events also interfered with social time

## 3. Detail to understanding programmatic elements

### 3.1. Program successes

Across 2018–2020, key B/C-Sharp elements established its overall success, leading to important findings related to improved dyad QOL as well as implications about community-based psychosocial programming. These included *an emphasis on mixed-methods program evaluation*, with qualitative data yielding the most valuable information, *coordination and collaboration with CPs*, and the *community-driven nature of this program*.

#### 3.1.1. Mixed/qualitative research processes

Throughout the program evaluation process, qualitative components (interviews and observations) most successfully engaged participants and generated valuable findings as CPs could actively facilitate these methods with PWDs. Furthermore, social hour/performance observations and CP interviews were often part of the “fun” of B/C-Sharp whereas PWDs found the RBANS stressful, making it harder to incentivize completion. Thus, we obtained qualitative data more easily *via* enjoyable processes, successfully generating valuable insights into dyads’ program experiences.

#### 3.1.2. CP engagement and buy-in

As the program developed and its complexity increased, it became clear that program success depended upon engaging CPs and showing them the value of participation. Efforts to introduce new evaluation procedures without first consulting CPs could result in implementation challenges, inconsistent data, and less effective programming. When CPs were excited about programming, they engaged with methods more consistently. For example, when feedback highlighted that symphony attendance could be difficult due to the late start times, our team created programming options with matinee (e.g., dance) or shorter performances (e.g., theater). Similarly, the introduction of saliva sampling was embraced by CPs and, in turn, PWDs, as the team made special materials to explain the collection process, its purpose, and why it mattered. Saliva sampling experienced high participation, which we largely attribute to our collaboration with CPs.

#### 3.1.3. Community-university collaboration

From its onset, B/C-Sharp was driven by a strong community-university partnership. Prior to program recruitment, the research team secured ticket donations for season performances from the local symphony, dance troupe, theater troupe, and performing-arts center. Community collaborators assisted with other aspects of the program. A local nonprofit functioned as the financial hub for donations and provided snacks for symphony performances. Other area nonprofits collaborated on recruitment and program enrollment. Ultimately, B/C-Sharp thrived because it was supported by multiple community organizations in addition to the interdisciplinary research team (Malinin et al., in press).

### 3.2. Program challenges

While B/C-Sharp had numerous successful components, it was not without challenges, which centered on *quantitative data procedures,* difficulties *enrolling and sustaining participation*, and overall *program complexity*.

#### 3.2.1. Quantitative cognitive data procedures

The primary quantitative evaluation outcome was cognitive performance as measured by the RBANS (Davalos et al., 2019). Participants found the RBANS frustrating and long. Additionally, dyads were usually separated during testing (though not in cases of emotional distress or advanced ADRD). To address these challenges, the research team began offering season pre−/post-test data collection events at separate dates/times from program events. While some participants did attend these events, they did not completely solve data collection challenges. Furthermore, participants would sometimes skip testing post-performance or arrive too late for testing pre-performance. As such, RBANS data was often incomplete.

#### 3.2.2. Quantitative physiological data procedures

Partway through the program, we sought to introduce activity tracking before, during, and after programming. As cognition and QOL can be improved with additional exercise and sleep quality, we explored what role physiological changes played in program outcome measures. A small number of dyads were invited to wear Fitbits to track movement and sleep quality for approximately 5 days before a program event through approximately 5 days afterward. Through the pilot program, it became clear that using Fitbits would not be feasible. Too many PWDs found the Fitbits distracting or annoying, often removing and losing them. For CPs, remembering to keep Fitbits charged, keeping track of their own Fitbit and their partners’, and managing the additional research appointments needed to download Fitbit data was too challenging. Thus, this procedure was abandoned.

#### 3.2.3. Participant recruitment and persistence

Some quantitative data difficulties involved the broader challenge of engaging a difficult-to-reach population. Across B/C-Sharp, missing data became a significant problem as individuals skipped performances, declined to participate in testing due to frustration or fatigue, or dropped out because of ADRD progression or other circumstances. Even for participants who persisted in B/C-Sharp across multiple years, missing data made it difficult to draw meaningful inferences. Program sample sizes were often too small to adequately power the more sophisticated analyses needed to account for the myriad factors that influence changes in cognitive performance or QOL over the duration of a nine-month program.

#### 3.2.4. Program complexity

A final challenge was program complexity. Coordinating multiple community-based programs with different evaluation components could overwhelm participants. Even seemingly simple tasks (like completing intake surveys) often resulted in incomplete data because these procedures interrupted event social time (a key benefit of program participation) and felt less meaningful to CPs. Additionally, the barrage of data collection procedures meant that dyads sometimes picked and chose which they wanted to do at any given event—further complicating the data collection process.

### 3.3. Key findings: quality of life

**Figure 2:**
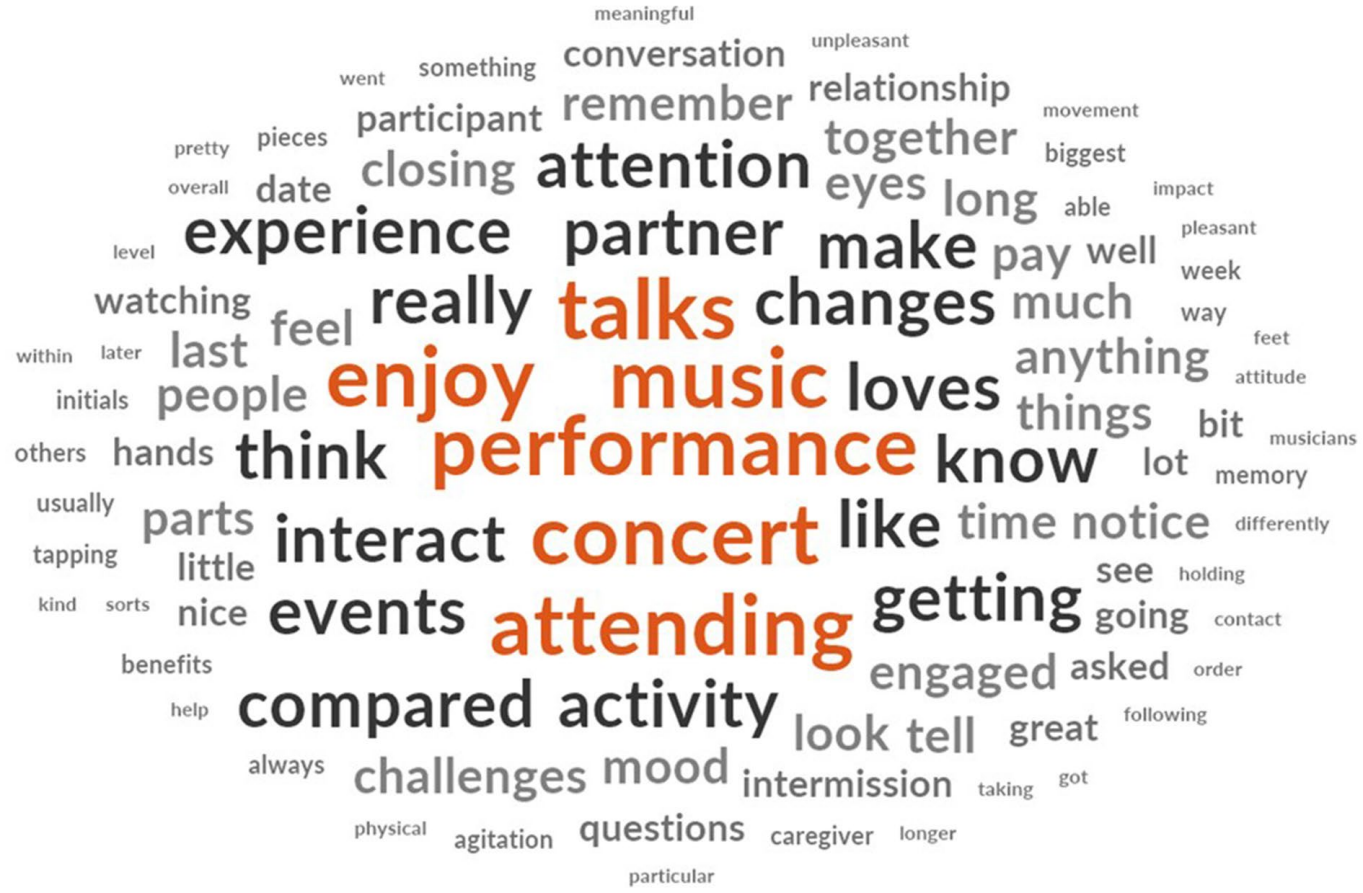
Word cloud generated from care-partner interviews.

Despite program challenges, we were able to draw upon qualitative data to assess behavioral, sociological, and QOL impacts of B/C-Sharp programming. In total, 47 interviews (symphony:33; dance:12; theater:2) were analyzed, and observational data from five events (October, November 2018; February, March, May 2019) was assessed. We used inductive analysis and visualizations using the NVIVO software program to identify themes (Figure 2). Key benefits included *positivity*, *engagement*, *social interactions and relationships*, and overall *improved wellbeing and QOL* due to program participation.^2^

#### 3.3.1. Positivity

Dyads used words like “enjoy,” “love,” “great,” “wonderful,” and other positive terms like “memorable” and “meaningful” to describe how they felt about the program. For example, a multi-year symphony CP articulated her consistently positive experiences: *“[It was] Good. Excellent, always is. I just enjoy the process, I enjoy thinking about it, I enjoy going there, I enjoy whatever degree of socializing can happen. I like it at all!”* Feelings of positivity were associated with several program aspects: going out for a special event with a loved one, the performance itself, seeing and interacting with others, and a positive change in mood.

Participants described how B/C-Sharp created a fun, safe space for dyads to have an evening out without overemphasizing the role of dementia in their lives. Direct observations noted a joyful atmosphere during the social hour, with participants smiling and joking. Participants also noted that the research team created a positive atmosphere, welcoming them warmly and making small talk. As a symphony CP described: *“It’s fun! You never know what’s going to happen.”* Observations noted that researchers used humor to reduce anxiety during cognitive assessments and saliva collection, and many participants described that the saliva collection was fun due to the joking around during collection.

Often dyads were motivated to participate because they had attended performing-arts programs together prior to the challenges of dementia. As one (dance) participant explained: *“It does make him happier to do something like this and go back to things he did when he was well. It’s a big relief for both of us, because life is really stressful because of his disease. This is a really nice getaway just to see him smile and have a good time. These kinds of things make him very happy.”*

For many participants, engaging in a social activity was a strong motivation for program participation. A symphony CP explained: *“[…] The best part was probably just being out for the evening. I think it’s just the socialization and getting to participate in something.”* Participants described enjoying socializing with others experiencing dementia, interacting with the research team (including student researchers), and connecting with the larger community.

Participation across all programs was associated with improved mood per interview, mood scale, and observational data. As a symphony CP shared: *“I just do not know how to explain. I mean she was just in a really more upbeat temperament when I took her home. I think she was a little scared when we first went because it was something she had never done before.”* Although most CPs described improved mood due to program activities, the research component caused stress for some, because it revealed how PWDs struggled with tasks that previously would have been simple. This was occasionally reflected in mood scales and observations noted a few participants appeared upset about quantitative assessments, typically RBANS. Usually a poor mood did not last, as one symphony CP observed: *“… it’s almost like she forgot about the testing by the end of [the evening]. She was just amazed about the ability of the musicians […] She was very impressed at their talent.”* CPs explained their mood was sometimes lower after the event in anticipation of returning to the daily challenges of living with dementia.

#### 3.3.2. Engagement

Participant engagement was high for all programs, with only two participants describing low engagement (one symphony PWD and one theater CP). Interview and observational data illustrated how PWDs were more alert during programming than they were with similar activities (e.g., watching a movie). CPs and researcher-observers reported PWDs watching performers intently, clapping hands and/or singing along, commenting about aspects of the performance, looking to other audience members for reactions, reading the program, and reminiscing after the performance. A theater CP explained: *“She was fully engaged and engrossed in the play and noticed other people around. She not only engaged with me and also the person seated on her other side […] she did not know her prior to the performance.”*

#### 3.3.3. Nurturing relationships

Nearly all CPs said B/C-Sharp improved their relationship with their PWD, with only two CPs noting no changes. Several CPs explained that B/C-Sharp helped them feel more connected with their partner, describing participation as a “date night” and reprieve from caregiving: *“Well, these days, we are at a point where I am definitely the caregiver. There were moments where I wasn’t in the role, which was nice […] it felt like an actual partnership”* (symphony CP). Many CPs described holding hands during the performance and reminiscing together afterwards.

B/C-Sharp also helped dyads create new relationships and allowed them to reconnect with acquaintances. Many CPs described becoming more isolated after their partner began exhibiting dementia symptoms, whereas B/C-Sharp helped them feel like a part of the community. A dance CP explained *“It’s hard—we are not a couple anymore like we used to be, so people do not include us anymore in those types of activities sometimes.*” Nearly all CPs described joy in connecting with others through the program. Observational data underscored dyads’ interactions, including conversations about the challenges of dementia and around shared interests, hobbies, etc. As a dance CP described: *“It wasn’t what we were expecting. We enjoyed getting out and being among other people. We both enjoyed it.”*

#### 3.3.4. Reminiscing

Many CPs described their surprise at what their partner could recall about B/C-Sharp events and how much they discussed their experiences together: *“Memory—he kept bringing it up. We talked about it all the way home, and the next day, he brought it up without even having the program in front of him. He brought it up without me mentioning it and said how great it was and what a different performance it was”* (dance CP).

#### 3.3.5. Other activities

Many CPs noted that B/C-Sharp renewed their interest in activities they used to do prior to their partner’s ADRD. A dance CP shared that he appreciated how the program changed their routine: *“[…] It’s just a great way to spend an afternoon, otherwise we would have just watched a football game on television, or she would have taken a nap.”* Several participants explained that B/C-Sharp gave them confidence to do more activities, and a few noted that it helped them understand the importance of staying active: *“[…] One of the things I learned the last year is how important it is to stay in the world. If something comes around, we do it”* (symphony CP).

### 3.4. Key findings: enriched environments

In addition to documenting B/C-Sharp’s effects on wellbeing, we sought to understand the role of the program’s enriched environments (visually, auditorily, and socially stimulating settings). Analysis of observational and interview data revealed three themes: *setting afforded social interactions, learning, and arts engagement*; *setting facilitated positivity and escapism*; and *spatial configuration created some challenges*.

#### 3.4.1. Affordances for social, educational, and arts engagement

The pre-performance social setting afforded opportunities for social connection. Observations noted that lines formed at the entrance to the event space as participants greeted researchers and each other. The refreshments and community table were hubs for social activities, enabling new participants to easily join conversations. Observers noted that participants moved between tables, forming and reforming small groups. Notably, observers did not record participants engaged in solitary activities (like browsing their phone) during social hours. Interviews also underscored the importance of the social space for connecting with others, as some CPs noted that socialization was more difficult on the few occasions where participants outnumbered the available seating.

The event space layout also enabled communication between participants and researchers, facilitating informal learning. The ticket and saliva collection tables were located near the front of the room, with researchers stationed at those tables engaging with participants and within earshot of those at the refreshment area. Participants shared that they felt comfortable chatting with both faculty and student researchers. Observations noted many participant-researcher conversations sharing research findings and available community resources.

Data revealed the role of others in the audience as a part of the B/C-Sharp experience. Observers recorded how participants would visually connect with other audience members during performances as a form of nonverbal communication, validating their emotional experience. They also chatted with study participants and other patrons during intermission and after the event, “checking in” to see if they had similar reactions. Many CPs cited this type of interaction as a program benefit and described how their PWDs initiated conversations about the performance with strangers, something they did not typically do.

#### 3.4.2. Atmosphere of “being away”

Observers noted the social atmosphere started out calm, with increased energy as the performance approached. Before walking to the performance, participants clustered together, talking in excited tones. This atmosphere of building anticipation created an engaging experience, with some specifically noting that in their interviews. During performances, participants were observed responding with emotion appropriate to the performance, including excitement (clapping, singing, etc.), surprise (raising eyebrows and looking around at others), and calm/relaxation (closing eyes). Observers noted that PWD showed signs of high engagement during performances and looked to other patrons to gauge their responses.

#### 3.4.3. Spatial configuration challenges

In interviews, CPs sometimes described issues related to physical obstacles. The most significant involved transportation and parking challenges. Some participants relied on a “Dial-a-Ride” service and were frustrated that they had to leave early to meet their pick-up window or were anxious that they might miss a ride. For those who drove, the number and location of parking spaces was difficult, as significant walking was required to get between parking, the social event space, and the performance space. Distance to the restrooms was also a challenge. Another common issue was the number and location of wheelchair seating in the performance halls. On a few occasions, CPs were unable to sit with their PWD who needed to remain in a wheelchair. The third significant theme related to challenges had to do with the timing of performances (mainly in the evening), with concerns their PWD would get tired or be at risk of “sunsetting behavior” that would not be conducive to enjoying an evening out.

## 4. Discussion

Our project’s aim was to co-design and evaluate performing-arts psychosocial programming for PWDs and CPs. Specifically, we explored whether performing arts could affect wellbeing for PWDs and CPs. Cognitive testing, while not statistically significant, showed a clear trend in improvement on total cognitive score and, more importantly suggested that brief neuropsychological testing is feasible in the context of arts-based, on-location settings. Qualitative data revealed that B/C-Sharp improved mood, memory, social relationships, and overall QOL. Our findings suggest that whichever types of performing-arts programming participants prefer (and find engaging) will likely prove the most beneficial for them. Additionally, architecture plays a role in psychosocial programming for PWD, including the need to go beyond universal design guidelines to create more inclusive spaces. Throughout our analyses, we identified clear successes and challenges in this complex, community-engaged program featuring a difficult-to-reach population. We now discuss the implications of our findings and recommendations for implementing similar programs in other communities.

### 4.1. Quality of life impacts of performing-arts programming

Our findings suggest that community performing-arts programming can improve PWD and CP sense of wellbeing and QOL by helping them experience “returning to normal” as a couple and engaging in a fun social experience without overemphasizing the role of dementia in their lives. B/C-Sharp also helped participants strengthen and expand their relationships. This is notable, as PWD and CPs’ networks often shrink post-ADRD diagnosis (Lindeza et al., 2020), and smaller networks can exacerbate feelings of isolation and burden for CPs (George et al., 2020). Our findings related to enhanced wellbeing were consistent across symphony, dance, and theater programs, indicating that similar programming focused on other performing arts might yield similar results. Additionally, CPs appreciated the opportunity to temporarily set aside their role and participate in a positive and supportive community. These outcomes generally support claims that dyads need opportunities to engage with others, and that these opportunities can be limited by the challenges of dementia (Gitte et al., 2019).

### 4.2. Enriched environments impacts of performing-arts programming

Our findings suggest that the physical environment can both enhance performing-arts programming *and* create barriers. Due to the passage of the Americans with Disabilities Act of 1990, U.S. buildings are constructed to universal design building codes. However, disability researchers argue that universal design is insufficient, suggesting instead an inclusive design approach. Coleman (1994) introduced the concept of inclusive design, arguing that “needs and abilities change throughout the life-course and that by taking account of this in the design process, […] environments can be improved for the majority of customers in ways that are not associated with negative perceptions of age or disability.” Performing-arts spaces are settings where people with disabilities, like ADRD, can connect with others. As such, these spaces are essential to creating age-friendly and dementia-friendly communities and providing the benefits of performing-arts programming to all.

### 4.3. Lessons learned

Throughout this project, we learned valuable lessons for those developing similar programming. The first lesson includes *connecting with and collaborating alongside CPs*. Throughout B/C-Sharp, we found CPs invaluable to the co-design and evaluation process. When CPs valued procedures, they participated eagerly and successfully persuaded PWDs to participate. For example, CPs helped us understand that methods that felt too “medical” detracted from the B/C-Sharp experience. As such, we worked with CPs to identify measures that would accomplish evaluation aims without removing the special-ness of the performing-arts experience. We advocate including CPs as valued participants *and* co-designers. CPs are highly knowledgeable about challenges and how PWDs respond to different circumstances; valuing their expertise builds capacity for better research and increases participant buy-in.

A second lesson includes *selecting and implementing evaluation measures carefully*. Throughout B/C-Sharp, we collected numerous forms of data to document program outcomes: short- and long-term cognitive performance (RBANS), mood, QOL, saliva samples, post-performance interviews, and observational data. Our intention was to have a thorough account of B/C-Sharp outcomes; the result was a patchwork collection of measures. Thus, we recommend choosing a few measures with dyads in mind (in collaboration with CPs) to ensure a more complete dataset toward more conclusive findings.

A final lesson learned is to *strike a balance between participant desires and research needs*. Throughout B/C-Sharp, dyads could be skeptical of procedures core to addressing research questions. Acknowledging these concerns while underscoring the measures’ value was essential. This involved communicating clearly and often with stakeholders about research goals and the value of participation. Creating a sense of value in participants (especially CPs) was key. As such, investigators should not automatically abandon measures met with hesitance from participants, instead working collaboratively with them to communicate their value, increase buy-in, and, if need be, identify alternatives.

### 4.4. Limitations and future directions

Despite its compelling findings, this project had limitations. First, participants were drawn from a community sample, resulting in a homogenous participant pool (i.e., predominately white, educated, middle class, from the same geographic region, etc.). Additionally, asking dyads to participate in an intervention over several months was challenging. ADRD progression can be unpredictable (Verdi et al., 2021), making it difficult for some dyads to commit to B/C-Sharp. As a result, some participants missed program events, resulting in missing data. Similarly, we were unable to maintain a waitlist control group because of study attrition. As such, in 2019–2020, we implemented a healthy control group (older adults without cognitive decline and not in a caregiver role). Unfortunately, the COVID-19 pandemic halted the program before we could collect sufficient data. Ultimately, challenges in participant recruitment/retention and the COVID-19 pandemic combined, making it difficult to draw conclusions from our quantitative data.

Given the significant burdens resulting from ADRD, there is a critical need to identify effective non-pharmacological, psychosocial interventions promoting cognitive health and wellbeing. Despite its limitations, our program points to how performing arts might lead this new research frontier. Contributions from B/C-Sharp include its innovative design and important findings. First, our approach was translational, informed by basic science, clinicians, and community agencies. Second, our approach was comprehensive, with interventions intentionally designed as integrated, community-based experiences that took advantage of the rich programming already available. Finally, our team developed an inventory of protocols for measuring physiological responses, social engagement, QOL, program experiences, and cognitive function that captured rich (qualitative and quantitative) outcomes helping us better understand key components in effective therapeutic applications of community performing-arts programming.

## Data availability statement

The raw data supporting the conclusions of this article will be made available by the authors, without undue reservation.

## Ethics statement

The studies involving human participants were reviewed and approved by Colorado State University Institutional Review Board. The patients/participants provided their written informed consent to participate in this study.

## Author contributions

LM, MF, and DD contributed to the program design and evaluation and to the manuscript creation, including concept, organization, writing, and editing. LM created Figures 1 and 2 and organized Table 1, with input from MF and DD. All authors contributed to the article and approved the submitted version.

## Funding

This project was supported by an award from the Research: Art Works program at the National Endowment for the Arts: grant# 1809975-38-18, along with funding from the Catalyst for Innovative Partnerships grant program through the Office of the Vice President for Research at Colorado State University and by the Columbine Health Systems Center for Healthy Aging (Pilot Funding for Innovative Research in Aging). Additional support was provided by the Fort Collins Symphony, Canyon Concert Ballet, Open Stage Theater, Fort Collins Lincoln Center, Banner Health, Community Foundation of Northern Colorado, Home State/Guaranty Bank, International Neuroscience Network Foundation, Kaiser Permanente, Rotary Club of Loveland, Sage Benefit Advisors, and individual community donors.

## Conflict of interest

The authors declare that the research was conducted in the absence of any commercial or financial relationships that could be construed as a potential conflict of interest.

## Publisher’s note

All claims expressed in this article are solely those of the authors and do not necessarily represent those of their affiliated organizations, or those of the publisher, the editors and the reviewers. Any product that may be evaluated in this article, or claim that may be made by its manufacturer, is not guaranteed or endorsed by the publisher.

## Footnotes

^1^Based on a power analysis calculated using G*Power statistical software, a sample of 45 dyads (or 15 dyads per intervention condition) would be sufficient to detect moderate-sized effects with 0.8 power for the RBANS assessments. Since the symphony program was ongoing, there were already dyads enrolled from the prior season who planned to continue in the program. We originally planned a waitlist control group; we were not able to recruit enough participants in the 18–19 season, so healthy controls were added in the 19–20 season.

^2^The first author analyzed the qualitative data through a process of inductive analysis in the NVIVO software application. Emergent themes were compared with qualitative findings from prior seasons of the symphony program (2015–16, 2016–17, and 2017–18), which were analyzed by another member of the research team (see Faw et al., 2021).

## References

[ref1] Association A. (2019). 2019 Alzheimer’s disease facts and figures. Alzheimers Dement. 15, 321–387. doi: 10.1016/j.jalz.2019.01.010

[ref2] Boots L. M. M. Wolfs C. A. G. Verhey F. R. J. Kempen G. I. J. M. de Vugt M. E. (2015). Qualitative study on needs and wishes of early-stage dementia caregivers: the paradox between needing and accepting help. Int. Psychogeriatr. 27, 927–936. doi: 10.1017/S1041610214002804, PMID: 25566686

[ref3] Bourne P. Camic P. Crutch S. Hulbert S. Firth N. Harding E. (2019). Using psychological and physiological measures in arts-based activities in a community sample of people with a dementia and their caregivers: a feasibility and pilot study. J. Ag. Stu. Ther. 1. doi: 10.16966/jast.102

[ref4] Buckley R. F. Sparks K. P. Papp K. V. Dekhtyar M. Martin C. Burnham S.. (2017). Computerized cognitive testing for use in clinical trials: a comparison of the NIH toolbox and Cogstate C3 batteries. J. Prev Alzheimers Dis. 4, 3–11. doi: 10.14283/jpad.2017.1, PMID: 29188853 PMC5726304

[ref5] Cawthon R. M. Smith K. R. O'Brien E. Sivatchenko A. Kerber R. A. (2003). Association between telomere length in blood and mortality in people aged 60 years or older. Lancet 361, 393–395. doi: 10.1016/S0140-6736(03)12384-7 12573379

[ref6] Coleman R.. (1994). The case for inclusive design-an overview. In Proceedings of the 12th Triennial Congress, International Ergonomics Association and the Human Factors Association, Canada.

[ref7] Damjanovic A. K. Yang Y. Glaser R. Kiecolt-Glaser J. K. Nguyen H. Laskowski B.. (2007). Accelerated telomere erosion is associated with a declining immune function of caregivers of Alzheimer’s disease patients. J. Immunol. 179, 4249–4254. doi: 10.4049/jimmunol.179.6.4249, PMID: 17785865 PMC2262924

[ref8] Dauphinot V. Delphin-Combe F. Mouchoux C. Dorey A. Bathsavanis A. Makaroff Z.. (2015). Risk factors of caregiver burden among patients with Alzheimer’s disease or related disorders: a Cross-sectional study. J. Alzheimers Dis. 44, 907–916. doi: 10.3233/JAD-142337 25374109

[ref9] Davalos D. B. Luxton I. Thaut M. Cross J. E. (2019). B sharp—the cognitive effects of a pilot community music program for people with dementia-related disorders. Alzheimers Dement. 5, 592–596. doi: 10.1016/j.trci.2019.08.004, PMID: 31650015 PMC6804583

[ref10] Faw M. H. Luxton I. Cross J. E. Davalos D. (2021). Surviving and thriving: qualitative results from a multi-year, multidimensional intervention to promote well-being among caregivers of adults with dementia. Int. J. Environ. Res. Public Health 18:4755. doi: 10.3390/ijerph18094755 33946957 PMC8125580

[ref11] Fink H. A. Linskens E. J. MacDonald R. Silverman P. C. McCarten J. R. Talley K. M. C.. (2020). Benefits and harms of prescription drugs and supplements for treatment of clinical Alzheimer-type dementia. Ann. Intern. Med. 172, 656–668. doi: 10.7326/M19-3887, PMID: 32340037

[ref12] George E. S. Kecmanovic M. Meade T. Kolt G. S. (2020). Psychological distress among carers and the moderating effects of social support. BMC Psychiatry 20:154. doi: 10.1186/s12888-020-02571-7, PMID: 32252700 PMC7137514

[ref13] Gitlin L. N. Winter L. Burke J. Chernett N. Dennis M. P. Hauck W. W. (2008). Tailored activities to manage neuropsychiatric behaviors in persons with dementia and reduce caregiver burden: a randomized pilot study. Am. J. Geriatr. Psychiatry 16, 229–239. doi: 10.1097/01.JGP.0000300629.35408.94, PMID: 18310553 PMC2803044

[ref14] Gitte R. Elisabeth Dalby K. Elisabeth Muth A. (2019). Working out availability, unavailability and awayness in social face-to-face encounters: the case of dementia. Discourse Stud. 21, 258–279. doi: 10.1177/1461445619829238

[ref15] Lindeza P. Rodrigues M. Costa J. Guerreiro M. Rosa M. M. (2020). Impact of dementia on informal care: a systematic review of family caregivers’ perceptions. BMJ Support. Palliat. Care. 1–12. doi: 10.1136/bmjspcare-2020-002242 33055092

[ref16] Malinin L. Cross J. E. Davalos D. B. Faw M. H. Wilhelm L. Lassell R. (in press). “Designing community arts engagement for people with dementia and their informal care partners” in Design for Dementia, Mental Health, and Wellbeing. eds. Niedderer K. Dening T. Holthoff V. (England: Routledge)

[ref17] Olsen C. Pedersen I. Bergland A. Enders-Slegers M.-J. Jøranson N. Calogiuri G.. (2016). Differences in quality of life in home-dwelling persons and nursing home residents with dementia – a cross-sectional study. BMC Geriatr. 16:137. doi: 10.1186/s12877-016-0312-4, PMID: 27400744 PMC4939817

[ref18] Panossian L. A. Porter V. R. Valenzuela H. F. Zhu X. Reback E. Masterman D.. (2003). Telomere shortening in T cells correlates with Alzheimer’s disease status. Neurobiol. Aging 24, 77–84. doi: 10.1016/S0197-4580(02)00043-X, PMID: 12493553

[ref19] Randolph C. Tierney M. Mohr E. Chase T. (1998). Randolph C, Tierney MC, Mohr E, Chase TN. The repeatable battery for the assessment of neuropsychological status (RBANS): preliminary clinical validity. J Clin Exp Neuropsychol 20: 310-319. J. Clin. Exp. Neuropsychol. 20, 310–319. doi: 10.1076/jcen.20.3.310.823, PMID: 9845158

[ref20] Seibert M. Mühlbauer V. Holbrook J. Voigt-Radloff S. Brefka S. Dallmeier D.. (2021). Efficacy and safety of pharmacotherapy for Alzheimer’s disease and for behavioural and psychological symptoms of dementia in older patients with moderate and severe functional impairments: a systematic review of controlled trials. Alzheimers Res. Ther. 13:131. doi: 10.1186/s13195-021-00867-8, PMID: 34271969 PMC8285815

[ref21] Sörensen S. Conwell Y. (2011). Issues in dementia caregiving: effects on mental and physical health, intervention strategies, and research needs. Am. J. Geriatr. Psychiatry 19, 491–496. doi: 10.1097/JGP.0b013e31821c0e6e, PMID: 21502853 PMC3774150

[ref22] Thomas P. O’Callaghan N. J. Fenech M. (2008). Telomere length in white blood cells, buccal cells and brain tissue and its variation with ageing and Alzheimer's disease. Mech. Ageing Dev. 129, 183–190. doi: 10.1016/j.mad.2007.12.004, PMID: 18242664

[ref23] U.S. Census Bureau (2020). Quick facts, Fort Collins City, Colorado. Available at: https://www.census.gov/quickfacts/fact/table/fortcollinscitycolorado/POP010220#POP010220

[ref24] Verdi S. Marquand A. F. Schott J. M. Cole J. H. (2021). Beyond the average patient: how neuroimaging models can address heterogeneity in dementia. Brain 144, 2946–2953. doi: 10.1093/brain/awab165, PMID: 33892488 PMC8634113

[ref25] Vermunt L. Sikkes S. A. M. van den Hout A. Handels R. Bos I. van der Flier W. M.. (2019). Duration of preclinical, prodromal, and dementia stages of Alzheimer’s disease in relation to age, sex, and APOE genotype. Alzheimers Dement. 15, 888–898. doi: 10.1016/j.jalz.2019.04.001, PMID: 31164314 PMC6646097

[ref26] Ward M. C. Milligan C. Rose E. Elliott M. Wainwright B. R. (2021). The benefits of community-based participatory arts activities for people living with dementia: a thematic scoping review. Arts Health 13, 213–239. doi: 10.1080/17533015.2020.1781217, PMID: 32552336

[ref27] World Health Organization (2022). Dementia. Available at: https://www.who.int/news-room/fact-sheets/detail/dementia

