# Supplementary material for: Performing arts as a non-pharmacological intervention for people with dementia and care-partners: a community case study
Source: Front Psychol. 2023 May 9;14:1149711. doi: 10.3389/fpsyg.2023.1149711 (PMC10204650; doi:10.3389/fpsyg.2023.1149711)
Supplement: Supplementary file 1 [file Data_Sheet_1.pdf]

Supplementary Material Appendix A: CP Phone Interview Questions

Participant #_________
Date of Phone Call: __________
Interviewer Initials__________

Caregiver Phone Interview Questions (within the week following the performance):

How was the experience overall? *Probe: What do you and your partner enjoy most about attending the performance? Anything you did not enjoy?*

Tell us about how you interacted with your partner during the concert. *Probes: Were you holding hands or making other physical contact during the performance? Did you talk about the music at intermission?*

Did the way you and your partner usually interact change at all before, during, or after the concert? *Probes: What changes happened? How long did these changes last? Were these changes pleasant or unpleasant? How long did these changes last?*

Did you interact with others at the concert? *Probes: Who did you interact with? Were these interactions meaningful? How did they make you feel?*

What sorts of activities do you and your loved one do together? How does attending the concert compare to these other activities? *Probes: How was their agitation level at the performance compared to other activities? How much did you talk about the experience later as compared to other activities?*

After the performance, did you talk with anyone about your experience? *Probes: Who did you talk to? What was the nature of your conversation? How did you feel during/after this conversation?*

How does attending these events affect your relationship with your partner? *Probes: Can you think of specific examples of how your relationship with your partner has improved/declined because of attending these events? Do these events help you feel more/less connected to your partner? Why do you think that?*

How engaged was your partner at the event? *Probes: What did their attention look like? When did they pay the most attention? Were they engaged in any movement during the concert/performance such as tapping their feet? Was your loved one watching the musicians/dancers/actors or closing their eyes? Do they close their eyes in order to pay attention to the music?*

Did you notice any changes in your loved one's mood, attitude, or memory after the performance? *Probes: Did you notice any one of the pieces of music having a particular impact on mood? Were they able to remember the performance longer than other events you attend?*

What would you say are the biggest benefits of attending these performances for both you and your loved one? What are the challenges?

Supplementary Material Appendix B: Direct Observation Field Note Guide

FIELD NOTES GUIDE

From USC Libraries Research Guides,
http://libguides.usc.edu/c.php?g=235034&p=1561924

Characteristics of field notes:

1. Be accurate
2. Be organized
3. Be descriptive
4. Focus on research problem
5. Record insights and thoughts

Guidelines for Descriptive Content:

1. Describe the physical setting.
2. Describe the social environment and the way in which participants interacted within the setting. This may include patterns of interactions, frequency of interactions, direction of communication patterns [including non-verbal communication], and patterns of specific behavioral events, such as, conflicts, decision-making, or collaboration.
3. Describe the participants and their roles in the setting.
4. Describe, as best you can, the meaning of what was observed from the perspectives of the participants.
5. Record exact quotes or close approximations of comments that relate directly to the purpose of the study.
6. Describe any impact you might have had on the situation you observed

Guidelines for Reflective Content:

1. Note ideas, impressions, thoughts, and/or any criticisms you have about what you observed.
2. Include any unanswered questions or concerns that have arisen from analyzing the observation data.
3. Clarify points and/or correct mistakes and misunderstandings in other parts of field notes.
4. Include insights about what you have observed and speculate as to why you believe specific phenomenon occurred.
5. Record any thoughts that you may have regarding any future observations.
